# Simultaneous Delivery of Wharton’s Jelly Mesenchymal Stem Cells and Insulin-Like Growth Factor-1 in Acute Myocardial Infarction

**Published:** 2018

**Authors:** Shahram Rabbani, Masoud Soleimani, Mohammad Sahebjam, Mohammad Imani, Azadeh Haeri, Ali Ghiaseddin, Seyed Mahdi Nassiri, Jalil Majd Ardakani, Maryam Tajik Rostami, Arash Jalali, Seyed Hossein Ahmadi Tafti

**Affiliations:** a *Research Center for Advanced Technologies in Cardiovascular Medicine, Tehran Heart Center, Tehran University of Medical Sciences,Tehran,Iran.*; b *Department of Hematology, Tarbiat Modares University, Tehran, Iran. *; c *Iran polymer and petrochemical institute, Tehran, Iran. *; d *Department of Pharmaceutics, School of Pharmacy, Shahid Beheshti University of Medical Sciences, Tehran, Iran .*; e *Biomedical Engineering Division, Chemical Engineering Department, Tarbiat Modares University, Tehran, Iran.*; f *Faculty of veterinary medicine, University of Tehran, Tehran, Iran.*

**Keywords:** Myocardial infarction, Stem cells, IGF-1, Angiogenesis, Cardiac function.

## Abstract

Wharton’s jelly mesenchymal stem cells (HWJMSCs) hold promise for myocardial regeneration, but optimal treatment regimen (preferably with a growth factor) is required to maximize functional benefits. The aim of this study was to explore the cardioprotective and angiogenesis effects of HWJMSCs combined with insulin-like growth factor-1 (IGF-1) in the treatment of acute myocardial infarction.

The hydrogel consisted of Polyethylene glycol (PEG) and hyaluronic acid was prepared and characterized with regards to rheology, morphology, swelling, degradation, and release behaviors. To examine *in-vivo* effects, the hydrogels containing HWJMSCs either alone (Cells/hydrogel group) or with IGF-1 (Cells/hydrogel/IGF-1 group) were intra-myocardially injected into a rabbit myocardial infarction model. *In-vivo *efficacy was evaluated histological, immunohistochemical, echocardiography, scanning electron microscopy, and SPECT analyses. Eight weeks after infusion, the Cells/hydrogel and Cells/hydrogel/IGF-1 groups exhibited significantly increased left ventricular ejection fraction by echocardiography. Percent of ejection fraction was respectively 18.5% and 40% greater than control (*P* < 0.01). Vascular density (CD31 positive cells) of both treatment groups were more than the control group and this superiority was more remarkable in Cells/hydrogel/IGF-1 group. Cells/hydrogel/IGF-1 group showed the least defect size in SPECT analysis. Combinatory therapy with HWJMSCs and IGF-1 may additionally improve cardiac function and promote angiogenesis.

## Introduction

Myocardial infarction (MI) is the major cause of morbidity, mortality, and disability worldwide despite considerable advances towards treatment options during the last four decades. Administration of thrombolytic agents, infarct size reduction strategies such as administration of nitrates and ß-blockers and surgery, treatment of potentially fatal arrhythmias, and percutaneous transluminal coronary angioplasty (PTCA) have greatly decreased mortality after MI. These developments have reduced the in-hospital mortality; however, heart failure remains the leading cause of morbidity and mortality in MI patients and a considerable number of survivors are disabled (1, 2). Therefore, research on novel and adjunctive therapies to limit infarct size and advance MI treatment is in great demand.

Insulin-like growth factor-1 (IGF-1) is a hormone with similar molecular structure to that of insulin produced primarily by liver. It has been widely recognized as an important regulator with cardioprotective effects (3). IGF-1 plays a critical role in survival, growth, and homeostasis of various cell types, including cardiac cells. Inhibition of apoptosis, augmentation of calcium signaling, and protection against necrosis are among intriguing functions of IGF-1 (4, 5). IGF-1 has also been reported to prevent necrosis of viable myocardium, to improve cardiac function, and to reduce long-term left ventricular dilation and remodeling (6, 7). 

Recent evidences have suggested that IGF-1 also has a remarkable angiogenesis effect (3, 8), which promotes neovessel formation from the endothelium of preexisting vasculature occurring in many physiological and pathological conditions (9, 10). Cardiomyocyte survival, growth, and function are largely dependent on angiogenesis and microvascular function. Angiogenesis is crucial in preventing the transition of acute MI to heart failure (11). Therapeutic angiogenesis has been strongly proposed as a novel therapy approach for ischemic heart disease (12, 13).

Stem cell therapy is another promising approach for cardiac regeneration after MI. Preclinical and clinical studies conducted in the last decade have shown encouraging results. Numerous challenges including retention of cell viability for clinical transplantation and lack of highly potent therapeutic cells may limit translation to the clinic. Mesenchymal stems cells (MSCs), originally isolated from the bone marrow, are multipotent stem cells. MSCs can differentiate into various cells including endothelial cells of blood vessels, smooth muscle cells, cardiomyocytes, hepatocytes, adipocytes, chondrocytes, nerve cells, and osteoblasts (14, 15). However, some reports show a proliferative effect of bone marrow MSCs on cancer cells (16). Moreover, in older patients experiencing MI, the patients’ bone marrow derived MSCs have reduced potency for differentiation and growth and are almost dysfunctional (17). In addition to bone marrow, Wharton’s jelly compartment of the human umbilical cord is another source of MSCs. These human Wharton’s jelly mesenchymal stem cells (HWJMSCs) retain their multipotent stem cell characteristics by having most of their embryonic stem cell as well as MSC markers in primary culture and early passages (18). They can differentiate into many desirable cells (including cardiomyocytes, endothelial cells, adipocytes), are available in large numbers, proliferative, immune-privileged, and well tolerated after transplantation. Considering their natural chemoattraction to the cardiac tissue, induction of angiogenesis by secretion of numerous pro-angiogenic factors, spontaneous differentiation into endothelial cells and cardiac myocytes, and ability to populate the ventricular myocardium (19-21), HWJMSCs may be proposed as a promising approach to stimulate myocardial regeneration in MI. Intracoronary infusion of HWJMSCs in patients with ST-elevation acute MI was safe and effectively improved myocardial viability and heart function (22).

Hydrogels based on hyaluronic acid, are useful for biocompatible and efﬁcacious medical therapies. Hyaluronic acid has an important role in tissue development, embryogenesis, and tissue healing (23). Many Hyaluronic acid - based hydrogels have been synthesized for drug and cell delivery (24). 

PEG is an ideal candidate to add to hydrogels because it is biocompatible and inert (25) and therefore the biochemical cues in the hydrogel remain the same. It is nontoxic, nonimmunogenic, attractive material to incorporate into ECM and allows nutrient and oxygen transport (26). In this study, addition of PEG to the hyaluronic acid increased the stiffness of the hydrogel.

To the best of our knowledge, no report is available regarding simultaneous delivery with IGF-1 and HWJMSCs for MI treatment. Concurrent administration of these two agents could synergistically improve cardiac function, enhance angiogenesis, induce myocardial regeneration, and exert further additive beneficial effects compared to either therapy alone. Therefore, we investigated the cardioprotective effects and angiogenesis capability of IGF-1 and HWJMSCs on a rabbit model of MI.

## Experimental

*Isolation and culture of HWJMSCs*

This experiment was approved by the Research Ethics Committee at Tehran University of Medical Sciences. Fresh human umbilical cords were obtained after birth with the written informed consent of the parents, and immersed in cold Hanksꞌ balanced salt solution (HBSS, Sigma). The mesenchymal tissue was then diced into small fragments of about 3–5 mm and cultured in Dulbeccoꞌs modified Eagleꞌs medium (DMEM)/F12 (Invitrogen) supplemented with 15% fetal bovine serum (FBS; Invitrogen), 1% penicillin, and streptomycin (Sigma). The cells were incubated at 37 °C in a humidified atmosphere of 5% CO_2_-95% air.

*Phenotype analysis of HWJMSCs*

The cells from passages 2 to 4 cultured in 24-well plates were used to measure alkaline phosphatase activity of HWJMSCs. After formation of colonies, the cultures were washed with phosphate buffered saline (PBS) and stained with an alkaline phosphatase kit (Sigma kit 86) according to the manufacturerꞌs instructions.

*Hydrogel preparation*

The hydrogel was composed of 30% w/v PEG (6 kDa) and 70% w/v hyaluronic acid (~50 kDa, Lifecore Biomedical). The hydrogel containing hyaluronic acid and PEG was prepared according to the method of Liu *et al* (27). Briefly, hyaluronic acid was completely dissolved in aqueous solution, followed by magnetically stirring. After being placed still at room temperature for 30 min, hyaluronic acid stirred vigorously. The resultant mixture was concentrated using PEG powder to get an ultimate concentration of 3.0% wt.

IGF-1 was dissolved in PBS (pH 7.4) to obtain a stock solution with a concentration of 1000 μg/ mL. Then, 0.1 mL of this solution (100 μg) was added to 0.9 mL of hydrogel solution. The IGF-1 concentration was designed to be 100 μg/mL of each injected hydrogel. After thoroughly mixing, the mixture was kept at 4 °C. Before injection, the mixture was transferred into a 37 °C water bath to obtain hydrogel containing IGF-1.

IGF-1 diluted 100X for each formulation before the release test but to achieve sufficient neovascularization for *in-vivo *study 100 µg of IGF-1 was added to the 0.9 mL of the hydrogel.

*Hydrogel characterization*

*Rheological measurements*

Rheological parameters were measured in triplicate at 25 °C, using a cone-and-plate instrument (Brookfield model DV-III, USA), equipped with a CP42 spindle. Rheological measurements were performed with increasing shear rates (0.3–210 rpm) to obtain plots of shear stress versus shear rate. 

*Interior morphology of hydrogel*

Freeze-dried hydrogel was carefully fractured and the interior morphologies were visualized using a scanning electron microscope (SEM, JSM-6380).

*Hydrogel swelling experiment*

Lyophilized hydrogels with equal weights (n = 3) were immersed in PBS of pH 7.4 at 37 °C with 100 rpm agitation and the water sorption was followed by gravimetry. At predetermined time intervals, the hydrogels were removed, surface-dried with the absorbent paper, and immediately weighed (Wt). The swelling (%) was calculated as follows:

Swelling (%) = (Wt-Wd) /Wd × 100%

where, Wt is the weight of the swollen hydrogels at time t and Wd is the weight of the lyophilized hydrogels.

*Hydrogel degradation experiment*

*In-vitro* degradation of the scaffold was evaluated at 37 °C in PBS at pH 7.4 by determining the weight loss according to the following equation:

Degradation (%) = (Mi-Mt) /Mi×100%

where, Mt is the weight of the dry hydrogels at time t and Mi is the initial weight of the dry hydrogels.

*Infrared (IR) spectroscopy*

The IR spectra of PEG, lyophilized hyaluronic acid, and PEG/ hyaluronic acid hydrogels were obtained using IR spectrometer (PerkinElmer 843). About 3 mg of the sample was mixed with KBr and the spectra were obtained in the wavenumber range of 4000–400 cm^−1^.

*In-vitro IGF-1 release*

IGF-1 loaded hydrogels were continuously stirred in PBS solution (pH 7.4) at 37 °C. At predetermined time points, the solutions were centrifuged at 10000 rpm for 5 min, and the amount of released IGF-1 was evaluated by ELISA.

*Cell viability*

HWJMSCs viability was assessed with the 3-(4,5-dimethylthiazolyl-2)-2,5-diphenyltetrazolium bromide (MTT, Sigma) assay. The MTT absorbance was read at different time intervals (1, 3, 5, 10, and 14 days) at 570 nm with 650 nm as the background absorbance using an ELISA plate reader (Expert 96, Asys Hitch, Ec Austria) and normalized to the absorbance measured at the beginning of study.

*MI Induction and HWJMSCs transplantation*

*Animals*

Male New Zealand white rabbits weighing between 2.0 and 2.5 kg, purchased from Razi Institute (Karaj, Iran), were used for this study. The animals were individually housed under controlled temperature (23 ± 2 °C) and a 12-h light/dark cycle with free access to food and water. All the experiments were performed according to the policies of the Institutional Animal Care and Use Committee at Tehran University of Medical Sciences in accordance with the NIH Guide for the care and use of laboratory animals (NIH publications No.8023, revised 1987).

*Cell preparation for transplantation*

10^6^ viable cells were injected intramyocardially into the infarcted region and its periphery at 4–5 sites (100 μL/point) using a 28G needle.

*MI model and treatment groups*

MI was induced by ligating the left coronary artery as previously described (28). Briefly, the animals were anesthetized with intramuscular injections of ketamine (50 mg/kg) and xylazine (5 mg/kg), intubated, and mechanically ventilated. Under sterile conditions, the heart was exposed via a left thoracotomy incision and the left anterior descending coronary artery (LAD) was ligated proximally with a 5-0 silk (Ethicon Inc., USA) suture. After the surgery, the rabbits were treated with analgesics and antibiotics. 

For proving MI, cardiac troponin T was measured before and one day after surgery. One hour after MI induction, the rabbits were randomly divided into 3 groups and were injected with: 1) PBS (control group); 2) HWJMSCs and empty hydrogel (Cells/hydrogel group); and 3) HWJMSCs and IGF-1 loaded hydrogel (Cells/hydrogel/IGF-1 group). Another 6 rabbits undergoing thoracotomy without coronary ligation were used as the sham group.

*Histopathological analysis*

Two months after injection, the animals were heparinized by intravenous injection of 500 U/kg and then sacrificed by an overdose of pentobarbital sodium. After heart removal and fixation in 10% buffered formalin, the left ventricle (LV) was sectioned into five equally spaced transverse slices from base to apex and paraffin-embedded. 6 µm thick serial sections were cut from each paraffin block and stained with hematoxylin-eosin (H&E). Inflammation was scored in a blinded fashion on a scale of 0–3.

*Immunohistochemical analysis*

Immunohistochemical staining was carried out on the tissue sections based on the labeled streptavidin-biotin method. Monoclonal antibodies against CD31 (DAKO, 1:100) was used and then the sections were treated for 30 min at room temperature with streptABComplex/HRP (DAKO, 1:400) according to manufacturer’s instruction. Subsequently, the slides were counterstained with Mayerꞌs Hematoxylin solution and CD31-positive capillaries were counted as previously described (29).

*Electron microscopic study*

Small proportions of infarcted anterior LV wall of each animal were removed immediately after scarification. Electron microscopic studies were performed on thin sections of glutaraldehyde-fixed cardiac tissues using JSM-6380 instrument to measure morphologically detectable injury. 

*Transthoracic echocardiography*

Transthoracic two dimensional echocardiography was performed at baseline as well as 1 and 60 days post-MI under light sedation (ketamine/xylazine) using a General Electric Vivid3 machine echocardiographic device equipped with a 7.5 MHz transducer. Left ventricular end-diastolic and end-systolic internal diameters (LVIDD and LVISD, respectively) as well as end diastolic and systolic volumes (EDV and ESV, respectively) were assessed. Fractional shortening (FS), FS% = (LVIDD − LVISD)/LVIDD ×100, cardiac output (CO), CO= Heart rate × (EDV-ESV), and left ventricular ejection fraction (EF), EF = (EDV-ESV)/ EDV ×100, were calculated. Echocardiographic measurements were made by an echocardiography specialist in a blinded fashion.

*In-vivo single-photon emission computed tomography (SPECT) imaging*

Under ketamine - xylazine anaesthesia, the animals were fixed in supine position at the center of the field of view of the SPECT camera. ^99m^Tc (185 ± 37 MBq) was administered via an ear vein. SPECT images were acquired 5 min after radiotracer injection to decrease background activity. Image acquisition was done using dual head Philips bright view gamma camera (Milpitas, CA 95035) equipped with high resolution parallel hole collimators. Acquisition parameters were 64 projections over 360, 30 s per projection with a matrix size of 256×256. SPECT images were reconstructed with iterative method using 2×8 or 2×16 iterations and subsets.

*Statistical analysis*

All statistical analyses were performed using SPSS software 16.0 (SPSS Inc., Chicago, IL, USA). The data were expressed as mean ± standard deviation (SD) and analyzed by t-test (for pairwise comparisons) or one-way ANOVA (for multiple comparisons) and the results were considered significant at *P* < 0.05.

## Results

*Rheological, swelling, and degradation behaviors of the hydrogel*

**Figure 1:**
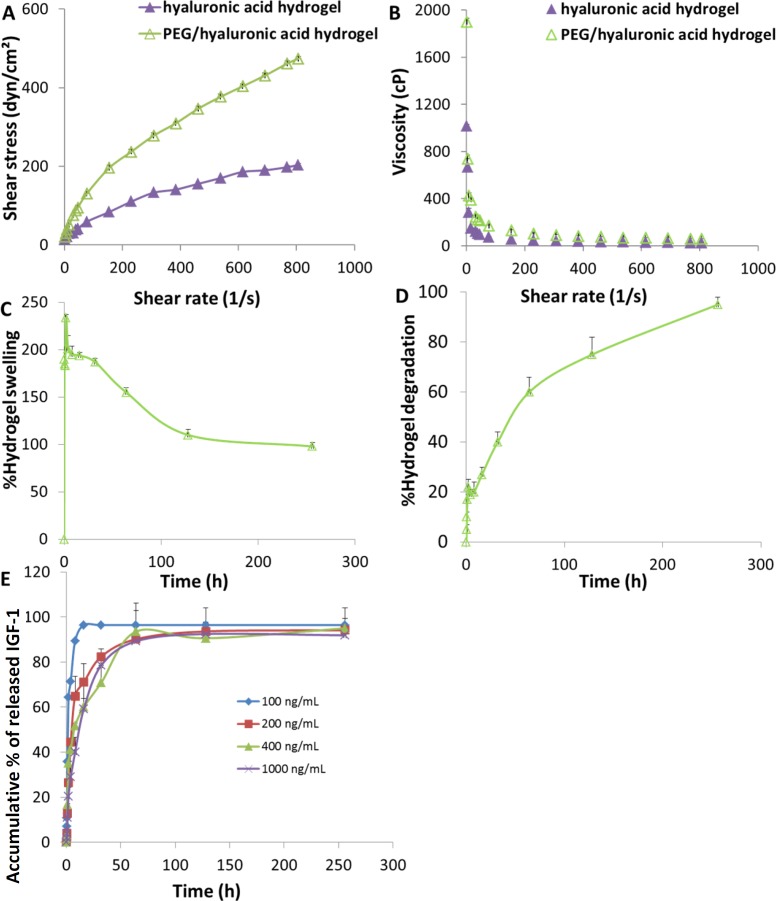
Hydrogel scaffold characterization. (A) Rheological profiles, (B) viscosity curves; (C) swelling and (D) degradation behaviors of the PEG/hyaluronic acid scaffold incubated at 37 °C in PBS (pH 7.4). (E) Release profiles of IGF-1 from the scaffolds loaded with different cargo concentrations in PBS (pH 7.4) (n = 3, mean ± SD).

The rheological behavior of an injectable hydrogel is a very important physical parameter that can be used to assess formulation consistency and to determine its stability. The studied hydrogels exhibited non-Newtonian behavior of pseudo-plastic flow (Figure 1A) i.e. starting to flow as the shear stress is applied and a decrease of viscosity with increasing shear rate. Moreover, the viscosity values of the hydrogels were not constant by increasing of the shear rate showing shear thinning behavior at high shearing rates (Figure 1B). As predicted, the lower viscosity values were observed with the hyaluronic acid hydrogel which was adequately adjusted by addition of PEG.

Swelling properties of cell scaffolds in aqueous media are crucial during cell culture which increase the pore size and facilitate cell infiltration in depth of the scaffolds as well as substance exchange. Swelling behavior of the hybrid scaffold is shown in Figure 1C. When immersed in aqueous media, the scaffold swelled immediately, which was clear from the profile in first 15 min of the study, reaching a plateau at 8 h with a swelling ratio of 195 ± 9% (Figure 1C). As time passed by, the swelling trend of the scaffold started to decrease. Weakening of the PEG/hyaluronic acid scaffold was observed after 16 h, which was almost the onset of degradation (Figure 1D). 

Scaffolds’ degradation rate should allow cells to adapt to *in-vi*vo environment and be regulated to match the extracellular matrix replacement rate while maintaining porosity and mechanical properties of the scaffold. The degradation behavior of the scaffold studied in PBS at pH 7.4 (Figure 1D) showed that the degradation rate was around 27% after 16 h incubation. From this time to the final time point, the degradation rate was more pronounced reaching almost complete degradation after 256 h.

*In-vitro IGF-1 release profile*

The release profiles of IGF-1 from the scaffold loaded with different concentrations of cargo ranging from 100 to 1000 ng/mL were studied using specific ELISA kit. As shown in Figure 1E, an initial burst release was observed in all profiles and about 20-60% of the IGF-1 released within the first 2 h., followed by a relatively controlled release profile. The loaded scaffolds released about 70-95% of IGF-1 within the first 48 h. Therefore, the IGF release must be localized and sustained in the target area.

*IR analysis of the hydrogel*

**Figure 2.**
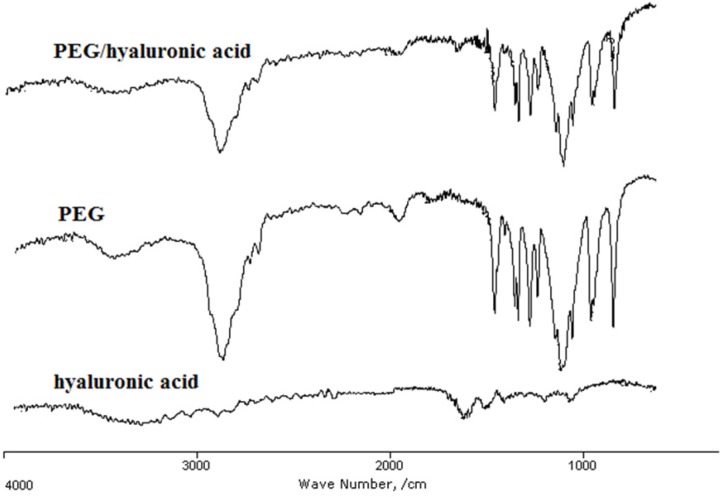
IR spectra of PEG, lyophilized hyaluronic acid, and PEG/ hyaluronic acid hydrogels

The presence of PEG and hyaluronic acid in the hydrogel scaffold and their interaction were studied using IR spectroscopy (Figure 2). In the IR spectra, the band 3400-3480 cm^-1^ represented the hydroxyl groups of hyaluronic acid and PEG. The strong peak at 1102 cm^−1^ in PEG and PEG/hyaluronic acid hydrogel is assigned to the ether bonds of PEG. The peaks observed at 1081 cm^−1^ and 1040 cm^−1^ as well as shoulder at 1480 cm^−1^ (associated with symmetric stretching of carboxylate anion) and the stronger band at approximately 1600 cm^−1^ (associated with asymmetric stretching of carboxylate anion) could be due to hyaluronic acid in the structure. The presence of both agents in the hydrogel scaffold was proved by the presence of all the characteristic peaks of PEG and hyaluronic acid in the PEG/hyaluronic acid hydrogel (Figure 2). The bands in the hybrid hydrogel were not significantly changed compared to the hyaluronic acid hydrogel indicating no major interaction.

*Internal morphology of the hydrogel*

**Figure 3.**
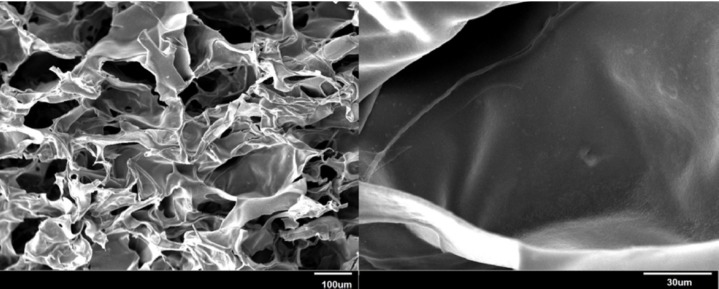
SEM micrographs of the internal structures of the hydrogel at two magnifications

Pore size and interconnectivity are important parameters in scaffolds. The morphological characteristics of the hydrogel were assessed using SEM. SEM micrographs of the lyophilized scaffold illustrated in Figure 3 showed distinct pores and inherent pore interconnectivity.

*Cell viability and proliferation*

**Figure 4:**
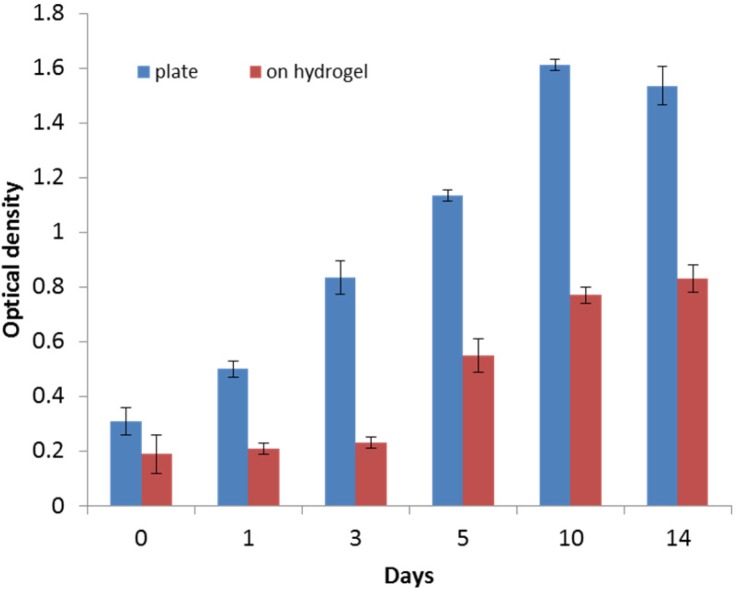
Cell viability evaluation by MTT assay. Formazan absorbance at 570 nm expressed as a measure of HWJMSCs viability cultured on the control surface and the hydrogel scaffold for a 14-day period (n = 3, mean ± SD

HWJMSCs were seeded and grown on the control surface (treated tissue culture) as well as on the PEG/hyaluronic acid scaffold. At different time points during 2 week period, cell proliferation was quantified using MTT assay. Monitoring the cells’ viability over time showed that the viability on the hydrogel generally increased (Figure 4). However, cells proliferated much faster on the control surface. After 3 days, the growth of cells on the hybrid scaffold significantly accelerated.

*Serum cardiac troponin T measurement*

MI was associated with cardiac necrosis evident by troponin release. Serum cardiac troponin T is routinely used as a humoral biomarker for cardiac injury. Measurement of serum cardiac troponin T was performed before surgery and 24 h after in all groups. Control and treatment groups exhibited a similar pattern of troponin T elevations (Table 1). Postoperative serum troponin T was significantly higher than preoperative levels (*P* value < 0.01) confirming cardiac injury.

*Histopathological analysis*

**Figure 5:**
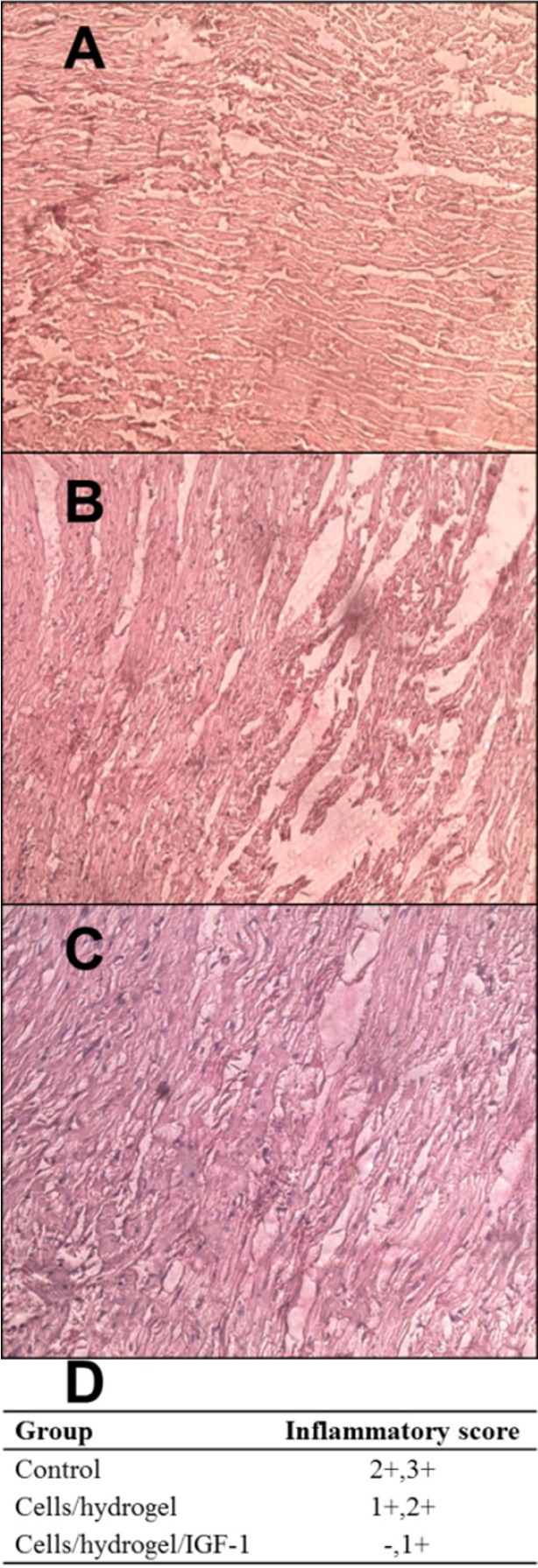
Representative images (×200) of H&E staining in the infarcted area of control (A), Cells/hydrogel (B), and Cells/hydrogel/IGF-1 (C) groups. (D) Inflammatory score in control and treated groups (n = 6

H&E staining was performed to observe the overall pathology of cardiac tissues (Figure 5). At day 60 post-injection, scar tissue consisting of fatty tissue and collagen was observed in the infarcted zone of all groups. Inflammation was scored to -, +, ++, and +++. Data are shown in Figure 5D. Control animals presented a severe inflammatory infiltrate mainly composed of granulocyte, monocyte, and lymphocytes (Figure 5A). In contrast, the analysis of inflammatory score of myocardial tissues from both treated groups revealed a significant inflammation decrease (Figures 5B and 5C). The myocardium of Cells/hydrogel/IGF-1 group showed a mild inflammation (Figure 5C).

*Immunohistochemical analysis*

**Figure 6:**
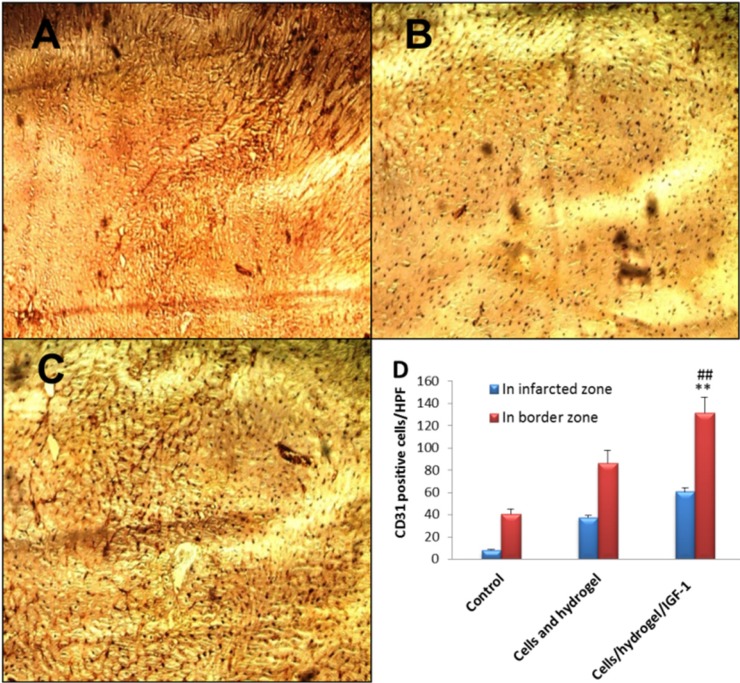
Representative images (×200) of anti-CD31 immunohistochemical staining in the infarcted area of control (A), Cells/hydrogel (B), and Cells/hydrogel/IGF-1 (C) groups. (D) The number of CD31 positive cells per hpf in control and treated groups (n = 6, mean ± SD). **P < 0.01 vs. control, ^##^*P *< 0.01 vs. Cells/hydrogel

14 days post MI, CD31-positive tubular structures (capillary density) were clearly greater in the infarcted as well as peri-infarcted zones of both Cells/hydrogel and Cells/hydrogel/IGF-1 groups than those in the control group (Figure 6). The microvessel density in the infarcted zone was 8 ± 1/high power field (HPF), 37 ± 2/HPF, and 61 ± 4/HPF for the control, Cells/hydrogel, and Cells/hydrogel/IGF-1 groups, respectively. The number of CD31-positive microvessels in the infarcted and peri-infarcted zones increased about 1.5-1.6 fold when IGF-1 was added to the treatment regimen (*P* value < 0.01). 

*Scanning electron microscopy analysis*

**Figure 7:**
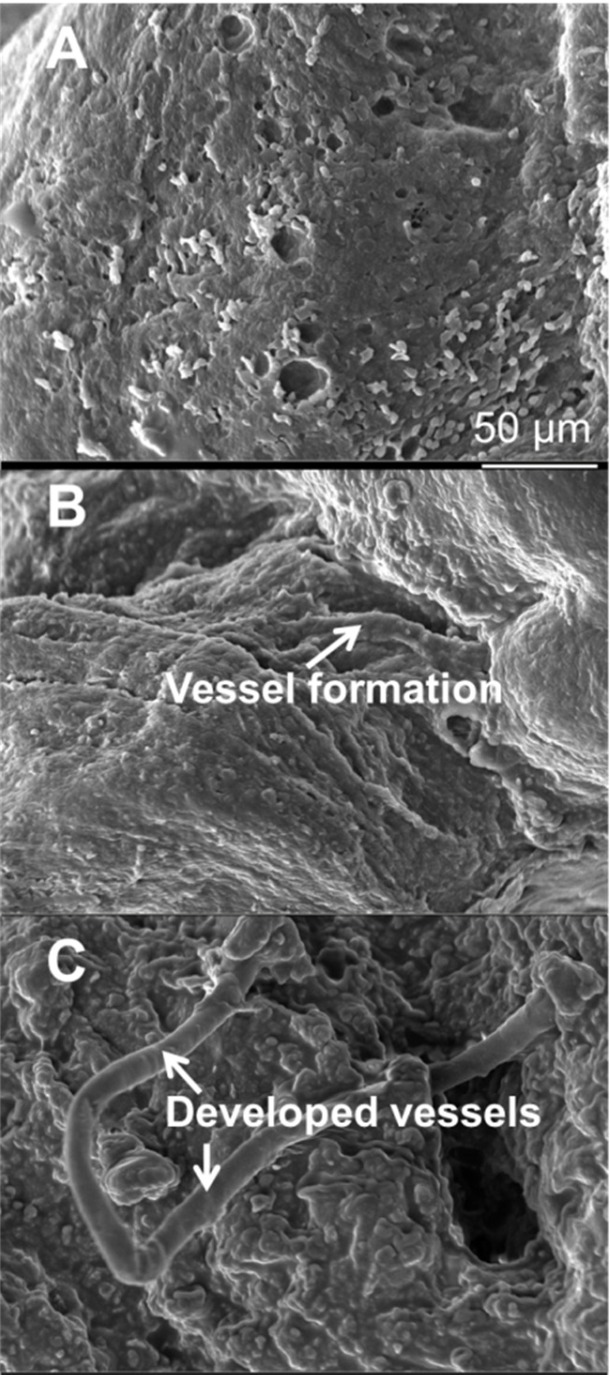
SEM images of infarcted heart of control (A), Cells/hydrogel (B), and Cells/hydrogel/IGF-1 (C) groups 8 weeks after MI induction

To observe presence of vasculature in the injured area, SEM analyses were performed at 60 days post MI. As the microscopy images were showed in the control group, no vessel and angiogenic elongation was observed in the infarcted myocardium area (Figure 7A). However, there were a few immature vessels in the cell group (Figure 7B) and many mature vessels were found in the IGF-1 group (Figure 7C).

*Cardiac function assessment*

The results of the echocardiographic measurements performed at baseline, 1 day postoperative, and 2 months postoperative are presented in Table 2.

Comparison of EF before and one day after surgery in all groups was statistically nonsignificant (*P* = 0.846). At 60 days post-surgery in control untreated animals, reduced anterior wall motion of the viable myocardium was demonstrated. A dilated LV cavity with thinning of the anterior wall was observed. A statistically significant improvement of EF was demonstrated with both Cells/hydrogel and Cells/hydrogel/IGF-1 therapies (*P* = 0.000). In both treated groups studied at day 60, diastole and systole LV diameter were significantly decreased, while fractional shortening and cardiac output were significantly improved, compared with respective measurements at day 60 from control untreated animals (all *P* values < 0.05). Significant differences in FS, CO, and EDV were observed between Cells/hydrogel and Cells/hydrogel/IGF-1 groups (*P* value < 0.01).

*SPECT analysis of myocardial perfusion*

**Figure 8.**
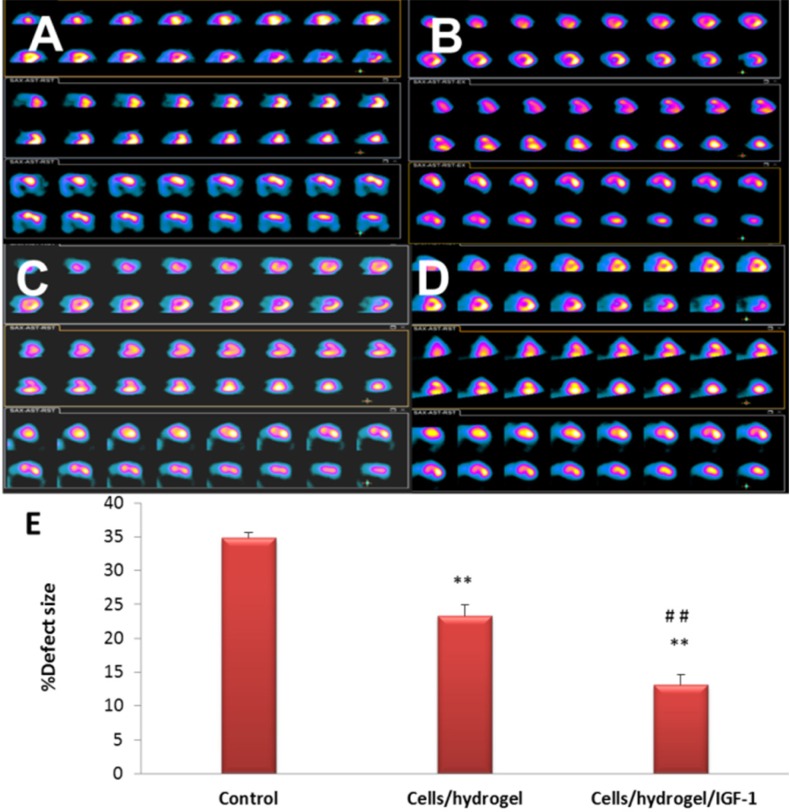
Representative SPECT images of sham (A), control (B), Cells/hydrogel (C), and Cells/hydrogel/IGF-1 (D) groups 8 weeks after surgery. (E) Defect size of myocardium (%) in different groups (n=6, mean ± SD). **P < 0.01 vs. control, ^##^*P* < 0.01 vs. Cells/hydrogel

SPECT measurements for the control, Cells/hydrogel, and Cells/hydrogel/IGF-1 groups were compared at 60 days post-MI (Figure 8). A significant perfusion defect was observed in the distal and anteroapical walls of the LV of control animals (MI with no treatment, Figure 8). 8 weeks after MI, total perfusion deficit was significantly reduced in the rabbits treated with HWJMSCs or HWJMSCs /IGF-1 (34.83 ± 0.75%, 23.33 ± 1.63%, and 13.16 ± 1.47% in the control, Cells/hydrogel, and Cells/hydrogel/IGF-1 groups, respectively). SPECT perfusion defect extent was significantly lower in both Cells/hydrogel and Cells/hydrogel/IGF-1 groups compared with the control group with respective p values of 0.04 and 0.01 (Figure 8D). Furthermore, perfusion defect extent was decreased in the Cells/hydrogel/IGF-1 group compared to the Cells/hydrogel group (*P* value < 0.01).

## Discussion

IGF-1 is one of the promising agents claimed to have cardioprotective effects. Davani *et al*. studied the effect of IGF-1 perfusion in a murine model of myocardial ischemia/reperfusion injury. The results revealed IGF-1 prevented reperfusion injury and maintained myocardial cellular integrity (30). In a pig model, a single, low-dose of IGF-1 administered locally after acute MI showed long-term benefits in infarction size as well as cardiac wall structure and function (7). IGF-1 activates IGF-1 receptor prosurvival signaling and improves cardiac function. This signaling pathway is antagonized by the mouse mast cell protease 4 (MMCP-4) which causes IGF-1 degradation. Tejada *et al*. (31) found that deletion of the MMCP-4 encoding gene markedly reduced late infarct size by inhibition of IGF-1 degradation and, consequently, diminished adverse structural remodeling and cardiac dysfunction. In spite of all these promising results, some studies indicated that growth hormones had no effect on improving cardiac function (32, 33), but still IGF-1 seems to be an encouraging growth factor to protect the myocardium. 

**Table 1:** Cardiac troponin T levels (ng/mL) in control and treated groups (n = 6, mean ± SD).

Group	Cardiac troponin T levels		*P* value
	Before MI	After MI	
Control	0.1 ± 0.0	8.2 ± 1.2	0.00
Cells/hydrogel	0.1 ± 0.0	9.5 ± 0.6	0.00
Cells/hydrogel/IGF-1	0.1 ± 0.0	9.3 ± 0.8	0.00

**Table 2:** Pre- and postoperative echocardiographic measurements in control and treated groups (n = 6, mean ± SD

	**Control**			**Cells/hydrogel**			**Cells/hydrogel/IGF-1**		
	**Preoperative**	**1 day after**	**60 days after**	**Preoperative**	**1 day after**	**60 days after**	**Preoperative**	**1 day after**	**60 days after**
EF %	68.50±1.04	34.66± 1.50	28.66 ± 0.51	70.00 ± 1.67	33.50 ± 2.25	47.16 ± 1.47	70.00 ± 1.41	31.83 ± 1.72	68.66 ± 6.62
FS %	33.33±2.73	22.33± 2.16	21.00 ± 2.60	32.66 ± 2.16	24.16 ± 0.98	27.16 ± 1.47	32.16 ± 2.48	21.83 ± 1.16	35.16 ± 3.31
CO lit	0.69±0.06	0.48± 0.14	0.38 ± 0.07	0.78 ± 0.10	0.55 ± 0.06	0.69 ± 0.04	0.70 ± 0.08	0.60 ± 0.12	0.90 ± 0.04
LVIDD mm	15.11±1.77	15.76±1.10	16.80±0.89	16.68±0.51	16.11±0.80	11.21±2.31	16.51±0.79	16.06±0.81	9.76±1.08
LVIDS mm	10.96±0.89	13.26±1.24	14.45±0.88	12.16±1.13	12.85±1.04	9.20±0.50	12.55±0.88	13.25±0.89	9.00±0.83
EDV ml	6.63±0.51	6.70±0.55	6.98±0.14	6.55±0.32	7.28±0.95	6.81±0.47	6.67±0.24	6.56±0.35	8.11±0.61
ESV ml	2.18±0.26	2.51±0.24	2.88±0.39	2.23±0.27	2.70±0.17	1.71±0.23	2.40±0.22	2.56±0.18	1.18±0.39

Another emerging approach in the treatment of cardiovascular diseases is stem cell transplantation. Several types of stem cells including embryonic stem cells, bone marrow stem cells, induced pluripotent stem cells, and MSCs have been explored as potential donor cells (34-37), but the ideal cell type has not yet been determined. HWJMSCs have a unique combination of favorite characteristics including high potential for cell differentiation, easy collection, lack of ethical concerns, and no immunogenic and tumorigenic properties that make them highly suitable for transplantation (38).

The strategy of using a combination of cells and angiogenic/growth factors provides greater advantages towards inducing myocardial regeneration following MI and improvement of the cardiac cell therapy efficacy. Padin-Iruegas *et al*. (39) showed the addition of IGF-1 delivery to cardiac progenitor cells reduced infarct size and improved the recovery of myocardial function and structure after MI more than cardiac progenitor cells and IGF-1 alone (39). Zhang *et al*. (40) explored effects of sustained co-delivery of IGF-1 and hepatocyte growth factor on transplanted bone marrow mesenchymal stem cells, for acute MI treatment. Combination of the growth factors significantly promoted the stem cells capacity for migration, differentiation, and lack of apoptosis (40). However, up to now, no study has addressed the co-delivery of HWJMSCs and IGF-1 which is the focus of current research.

Hyaluronic acid scaffolds, promotes high cell adhesion and proliferation, low apoptosis, and regulated cell differentiation (41), whereas PEG properly adhere to the surrounding tissue and can form the extracellular matrix and scaffold in situ(42). There are reasons for which hyaluronic acid could be combined with other polymers for drug delivery: biodegradation of the hyaluronic acid, formation of self-assembled systems, prolonged plasma half-life, improved solubility, and improved pharmacokinetic features of the resulting compound (43).

For intramyocardial co-administration of IGF-1 and HWJMSCs, IGF-1 was loaded into PEG/hyaluronic acid hydrogel scaffold. The IGF-1 loaded hydrogel was characterized regarding swelling, degradation, cargo release behaviors, and internal morphology.

Swelling of the polymers occurs following the liquid phase diffusion into the hydrogel mass. The scaffold swelling property mainly depends on microstructure of scaffold, free-volume among chains, polymer hydrophilicity, and variable motion of the polymer chains (44-46). Due to abundant number of hydrophilic groups of PEG and hyaluronic acid such as carboxyl, amino, and hydroxyl groups, the scaffold showed good water uptake. By maximizing surface area/volume ratio and increase of the pore sizes, swelling facilitates infiltration of cells into the 3D structure of scaffolds and exchange of cell nutrients and metabolites within the hydrogel (46, 47).

Degradation of scaffolds is a crucial parameter in tissue engineering. Ideally, by formation of new tissue, the scaffolds should degrade in a controlled manner (48). Furthermore, the degradation products should be biocompatible and non-toxic (49). The degradation of implants is mainly through hydrolyzation in lysozyme and the degradation rate is inversely related to the crystallinity degree (50, 51). Therefore, the enhanced degradation of the PEG/hyaluronic acid scaffold could be explained by the easy adsorption and penetration of water molecules into the scaffold facilitated by the large number of hydrophilic groups.

As shown in Figure 1E, the *in-vitro* IGF-1 release showed biphasic release pattern. The initial burst release of IGF-1 within 24 h following MI could exert as a strong immediate pro-survival signal to reduce cell apoptosis and rescue the remaining functional cardiomyocyte after the ischemic event. Continuous sustained release of IGF-1 can also trigger processes such as angiogenesis induction required at subacute stages of infarct repair. This pattern of sequential IGF-1 delivery can increase scar thickness, reduce scar fibrosis, prevent infarct expansion, and promote angiogenesis and vascular maturation (52). 

Pore size and interconnectivity that can be observed by SEM (Figure 3) are important parameters in scaffolds because they play roles in cell ingrowth, diffusion of nutrients to accommodate cells and guide their growth, and subsequently tissue regeneration success (53, 54).

Before cell transplantation, one should ensure from the cell viability within the scaffold. The MTT assay (Figure 4) suggested that HWJMSCs can be cultured and proliferated on the scaffold as well as the culture plate but the proliferation rate was decreased during the first 48 h in the scaffold. We continued the culture up to 14 days to ensure the viability and proliferation of the cells. 

Following characterization of the scaffold and confirming the cell viability and proliferation capability on the scaffold, the efficacy of the controlled delivery system was evaluated in the rabbit model of acute MI. MI was induced by ligation of left anterior descending coronary artery. To confirm the MI in the animal model, serum cardiac troponin T concentrations were measured. Troponin is a diagnostic and prognostic humoral biomarker for cardiac injury. Elevation of troponin levels take 4 to 12 h after myocardial necrosis, peaking at 12 to 48 h from ischemia onset (55). Cardiac troponin T is a superior and sensitive humoral biomarker of myocardial damage in laboratory animals. All groups showed significant elevation of serum cardiac troponin T (0.1 ng/mL perioperative versus 8 ng/mL 24 h postoperative, Table 1). Following MI induction, the animals were randomly divided into 3 groups: 1) Control group receiving no treatment, 2) Cells/hydrogel group receiving HWJMSCs and empty hydrogel, and 3) Cells/hydrogel/IGF-1 receiving HWJMSCs and IGF-1 loaded hydrogel. Efficacy of the treatment strategy was evaluated by histopathological, IHC, echocardiographic, SPECT, and SEM analyses.

Histopathological analysis of heart sections revealed that the inflammatory severity of lesions in rabbits was significantly modified by Cells/hydrogel/IGF-1treatment. The number of CD31-positive vessels as an indicator of neovascularization in both Cells/hydrogel and Cells/hydrogel/IGF-1 groups was higher than in the control group. This value was greater following IGF-1 administration suggesting that co-delivery of HWJMSCs and IGF-1 has the greatest potential to promote neovascularization.

To determine whether HWJMSCs /IGF-1 transplantation can reduce the myocardial ischemia severity and improve myocardial perfusion and function, SPECT and echocardiography were used 8 weeks after treatment. In fact, Cells/hydrogel/IGF-1 group exhibited better regional perfusion, smaller infarct size, and improved wall motion coordination based on the 99mTC-SPECT scans and echocardiography analyses. In SEM images, mature formation of vessel can be observed in IGF-1 group compared with the Cells/hydrogel group that only immature vessels formed.

## Conclusion

In the present study, we demonstrate that the co-administration of IGF-1 and HWJMSCs enhanced angiogenesis in the infarct as well as the border zones, and consequently resulted in thicker scars with reduced fibrosis. It is also important to point out the improvement of EF and reduction of inflammation with this new co-administration strategy. All of these findings suggest that therapy outcome of cardiac tissue engineering and regeneration medicine is highly dependent not only on choosing an appropriate cell type, but also on use of a growth factor preferably with angiogenesis-promoting capability. Our study suggests that both preclinical and clinical studies may benefit from co-delivery of growth factors and cells.
